# 供者CD34^+^细胞纯化输注挽救治疗原发性骨髓纤维化异基因造血干细胞移植后移植物功能不良3例

**DOI:** 10.3760/cma.j.cn121090-20240117-00029

**Published:** 2024-08

**Authors:** 海霞 石, 慧霞 刘, 道林 魏, 骏 朱, 珊 邵, 瑛 蒋, 椿 王, 初娴 赵

**Affiliations:** 上海闸新中西医结合医院血液科，上海 200443 Department of Hematology, Shanghai Zhaxin Traditional Chinese and Western Medicine Hospital, Shanghai 200443, China

## Abstract

回顾性分析上海市闸新中西医结合医院2020年至2023年行异基因造血干细胞移植（allo-HSCT）后发生移植物功能不良并接受供者CD34^+^细胞纯化输注挽救治疗的3例原发性骨髓纤维化患者，其中男2例，女1例，中位年龄68（39～69）岁。启动供者CD34^+^细胞纯化输注距allo-HSCT的中位时间为83（56～154）d。输注的纯化供者CD34^+^细胞中位数7.67（7.61～9.06）×10^6^/kg，CD34^+^细胞纯度97.76％（96.50％～97.91％），回收率70％（42％～75％），2例获得血液学恢复。3例患者均未观察到急性GVHD，1例发生口腔慢性GVHD（中度）。供者CD34^+^细胞纯化输注治疗原发性骨髓纤维化allo-HSCT后移植物功能不良有效且未观察到严重的急慢性GVHD。

## Abstract (en)

A retrospective analysis was conducted on three patients with primary myelofibrosis who underwent allogeneic hematopoietic stem cell transplantation（allo-HSCT）at Shanghai Zhaxin Traditional Chinese and Western Medicine Hospital from 2020 to 2023. They subsequently developed poor graft function. The patients received selected donor CD34^+^ cell boosts as salvage therapy. There were two male patients and one female patient, with a median age of 68（39–69）years. The median time from allo-HSCT to the selected donor CD34^+^ cell boost was 83（56–154）days. The median infusion of selected donor CD34^+^ cells was 7.67（7.61–9.06）×10^6^/kg, with a CD34^+^ cell purity of 97.76％（96.50％–97.91％）and a recovery rate of 70％（42％–75％）. Hematological recovery was achieved in two cases. No acute GVHD was observed in any of the three patients. One case of moderate oral chronic GVHD was noted. Selected donor CD34^+^ cell boosts for the treatment of poor graft function after allo-HSCT in primary myelofibrosis was effective and no severe acute or chronic GVHD was observed.

异基因造血干细胞移植（allo-HSCT）是唯一可能治愈原发性骨髓纤维化（PMF）的治疗方法。然而，由于PMF患者脾脏显著增大、骨髓造血微环境差、频繁输血导致铁过载及患者年龄较大等因素，发生植入失败和移植物功能不良（PGF）的概率高于接受allo-HSCT的其他血液疾病患者，非复发死亡率较高[1]。针对PGF的治疗尚无标准方案，可以尝试的治疗方法有脾脏切除、调整免疫抑制药物剂量、应用造血生长因子、输注间充质细胞、供者淋巴细胞输注、供者CD34^+^细胞纯化输注、二次移植等。供者CD34^+^细胞纯化输注是目前治疗PGF的有效方法，与其他治疗手段相比，CD34^+^细胞纯化输注能帮助患者恢复造血，且不增加移植物抗宿主病（GVHD）和疾病复发风险[2]。但是CD34^+^细胞纯化输注应用于PMF患者的经验有限，国内尚无相关报道。本中心应用供者CD34^+^细胞纯化输注治疗3例移植后发生PGF的PMF患者，报道如下。

## 病例与方法

1. 临床资料：回顾性分析2020年至2023年在上海闸新中西医结合医院接受allo-HSCT且移植后发生PGF并接受供者CD34^+^细胞纯化输注挽救治疗的PMF患者3例。造血干细胞来源：1例为中华骨髓库HLA 10/10相合无关供者（MUD），另2例为亲缘半相合供者，均为年轻供者。例1，男，39岁，无可触及的脾脏肿大，移植前骨髓原始细胞比例65％，MF-2级（欧洲骨髓纤维化分级共识标准），供者特异性抗体（DSA）DQ和DR位点弱阳性，供受者ABO次要血型不合。例2，女，68岁，脾脏中度肿大，移植前MF-3级，供受者ABO主要血型不合。例3，男，69岁，脾脏重度肿大，移植前MF-3级，供受者血型相同。3例患者移植前预处理方案均为以全身放疗（TBI）联合塞替派+白消安+氟达拉滨（TBF）为基础的清髓方案，具体为：TBI 3 Gy，−7 d；氟达拉滨30 mg/m^2^，−6 d～−2 d，静脉输注；白消安3.2 mg/kg，−6 d～−5 d，静脉输注；塞替派5 mg/kg，−4 d～−3 d，静脉输注；阿糖胞苷1 g/m^2^，−6 d～−2 d，静脉输注。GVHD预防方案：兔抗人胸腺细胞免疫球蛋白2 mg/kg，−4 d～−2 d，静脉输注；环孢素A 2 mg/kg，−2 d开始，静脉输注，目标药物浓度200～300 ng/ml，或他克莫司0.02 mg/kg，−2 d开始，静脉输注，目标药物浓度6～8 ng/ml；甲氨蝶呤15 mg第1天，10 mg 第3天，10 mg第6天，静脉推注。3例患者回输的供者CD34^+^细胞数量分别为9.47×10^6^/kg、10.64×10^6^/kg和10.32×10^6^/kg。

2. 定义及疗效评价标准：PGF定义为供者造血干细胞输注28 d后，供者细胞呈完全嵌合状态且无重度GVHD，排除原发病复发、病毒感染及药物相关骨髓抑制等情况下，患者外周血提示两系或三系细胞减少，且需要依赖红细胞和（或）血小板输注及粒细胞集落刺激因子（G-CSF）治疗[3]。

CD34^+^细胞回收率（％）＝纯化分选后CD34^+^细胞数/动员后采集的CD34^+^细胞数×100％。

疗效评价标准：血液学恢复（HR）：ANC>2×10^9^/L，HGB>100 g/L，PLT>100×10^9^/L。血液学改善（HI）：ANC>1.5×10^9^/L，HGB>80 g/L，PLT>30×10^9^/L，摆脱输血依赖。无效（NR）：CD34^+^细胞纯化输注后仍需要依赖集落刺激因子和（或）成分输血支持。

3. 供者CD34^+^细胞采集和纯化方法：使用G-CSF 5～10 µg·kg^−1^·d^−1^进行供者外周血造血干细胞动员，于第5天采集供者外周血干细胞。应用CliniMACS Plus系统（德国 Miltenyi Biotec公司产品）对采集到的干细胞进行CD34^+^细胞分选，采用流式细胞术检测终产品CD34^+^细胞数值及纯度，计算得出回收率。

4. 供者CD34^+^细胞输注：纯化的供者CD34^+^细胞即刻全部输注给患者，输注前不进行预处理，输注后可应用G-CSF、TPO受体激动剂、促红细胞生成素、成分输血支持治疗等。

5. 随访：所有患者定期门诊及住院随访，随访内容包括临床有无急慢性GVHD表现、外周血象恢复情况、原发病评估及供受者嵌合状态，截至2023年11月30日，中位随访时间为allo-HSCT后15（4.5～22）个月。CD34^+^细胞纯化输注治疗后的生存时间定义为自CD34^+^细胞纯化输注至随访截止或死亡的时间。

6. 统计学处理：本研究采用描述性统计分析，CD34^+^细胞采集量、输注量等用中位数（范围）描述，CD34^+^细胞回收率、纯度用百分比描述。3例患者的随访和生存时间用中位数（范围）描述。

## 结果

1. 供者CD34^+^细胞采集和纯化结果：G-CSF动员后采集的供者CD34^+^细胞中位数为12.87（10.20～17.80）×10^6^/kg。CliniMACS PLUS系统分选后CD34^+^细胞中位数为7.67（7.61～9.06）×10^6^/kg。CD34^+^细胞纯度为97.76％（96.5％～97.91％），回收率为70％（42％～75％）。

2. PMF患者纯化CD34^+^细胞输注结果、疗效、GVHD及生存情况：CD34^+^细胞纯化输注治疗前，3例患者均已撤停环孢素A/他克莫司等免疫抑制药物，达到供者细胞完全嵌合状态。CD34^+^细胞纯化输注治疗后，3例患者中2例患者获得HR，1例患者早期死亡。获得HR的2例患者，CD34^+^细胞纯化输注前均粒系植入，红系及血小板植入不良，CD34^+^细胞纯化输注后红细胞恢复时间分别为输注后15 d和35 d，血小板恢复时间分别为输注后11 d和92 d。截至目前，2例患者均无复发生存，CD34^+^细胞纯化输注后生存时间分别为20个月和10个月（表1）。其中例1无急慢性GVHD表现；例2无急性GVHD（aGVHD）表现，于CD34^+^细胞纯化输注后195 d出现口腔慢性GVHD（cGVHD）（中度），予西罗莫司后症状缓解。例1 CD34^+^细胞纯化输注后无病毒感染。例2 allo-HSCT前即存在人类疱疹病毒6型（HHV-6）血症，经抗病毒治疗后转阴，CD34^+^细胞纯化输注后有巨细胞病毒（CMV）、HHV-6血症，经抗病毒治疗后均转阴。1例死亡患者CD34^+^细胞纯化输注后ANC>1.0×10^9^/L，但至输注后2个月HGB及PLT仍无改善，输血依赖，后期发生重症感染、多器官衰竭死亡。

**表1:** 3例原发性骨髓纤维化患者纯化CD34^+^细胞输注（SCB）情况及疗效

例号	性别	年龄（岁）	SCB前血常规			SCB前供受者细胞嵌合状态	SCB距移植时间（d）	纯化CD34^+^细胞输注量（×10^6^/kg）	SCB后3个月疗效	急慢性GVHD	SCB后生存时间（月）
			WBC（×10^9^/L）	HGB（g/L）	PLT（×10^9^/L）						
1	男	39	5.50	54	10	XY-FISH 1000/1000	56	7.67	HR	无	20
2	女	68	4.12	58	15	XY-FISH 1000/1000	154	14.03	HR	中度cGVHD	10
3	男	69	0.56	43	5	STR-BM 99.63%	83	9.06	NR	无	2

**注** HR：血液学恢复；NR：无效；cGVHD：慢性移植物抗宿主病

## 讨论

allo-HSCT是PMF患者唯一有望获得治愈的治疗手段。然而，植入失败和PGF仍是骨髓纤维化（MF）患者移植后需要面临的重要问题[4]。Gambella等[5]报道，接受单倍体造血干细胞移植的49例MF患者中，9例（18％）发生PGF。Alchalby等[6]报道了单中心100例MF患者，经减低剂量预处理后行allo-HSCT，PGF发生率为17％。目前已知的可能导致PGF的原因有：高龄受者或高龄供者；受者移植前原发病治疗NR；移植前存在DSA、铁过载、MF；输注的造血干细胞数量不足；移植前的预处理方案和移植后的免疫抑制方案；移植后发生GVHD或病毒感染（如CMV、HHV-6、人类细小病毒B19）等。移植前脾脏重度肿大（脾脏边缘达左肋缘下≥10 cm）的MF患者发生PGF的可能性更大，和无重度脾大患者相比，两组PGF的发生率分别为26％和13％（*P*＝0.13）。更值得注意的是，移植后30 d脾脏仍显著肿大者PGF发生率为33％，高于对照组的12％（*P*＝0.05）[6]。本文报道的3例PMF患者的供者均年轻，allo-HSCT时输注的供者外周血CD34^+^细胞数量均接近10×10^6^/kg。例1无脾大，但移植前骨髓原始细胞比例65％，DSA DQ和DP两个位点呈弱阳性，可能是其发生PGF的原因。例2高龄、持续存在HHV-6血症、脾大、供受者ABO主要血型不合；例3高龄、患病时间长、脾脏重度肿大，人类细小病毒B19感染，可能是其发生PGF的原因。

PGF对患者预后的影响主要表现为血细胞减少导致的临床并发症，感染和出血是导致患者死亡的主要原因。Gambella等[5]报道的9例PGF患者中，6例（66％）死亡，均为非复发死亡。Alchalby等[6]的研究结果显示，移植后发生PGF的17例患者中，死亡2例，死因均为真菌感染。PGF组患者3年预估总生存率不劣于无PGF组（66％对64％，*P*＝0.74），PGF组患者的生存获益是由于有效的治疗措施，如CD34^+^细胞纯化输注挽救治疗。北京航天中心医院王静波教授团队应用纯化的供者CD34^+^细胞输注治疗单倍体造血干细胞移植后PGF的12例患者，10例造血恢复，但该组患者中无MF病例[7]。国外应用该项技术治疗PGF的报道多为小样本研究，一项荟萃分析纳入7项研究共209例接受CD34^+^细胞纯化输注治疗的PGF患者，其中MF患者仅31例，未对其进行生存分析[2]。本文应用CD34^+^细胞纯化输注挽救治疗allo-HSCT后PGF的PMF，3例患者中，2例治疗有效，分别于CD34^+^细胞纯化输注后15 d和46 d摆脱输血依赖，输注后3个月获得HR，为PMF移植后PGF的治疗提供了临床经验。

CD34^+^细胞纯化输注治疗后造血仍未恢复的患者预后很差，Cuadrado等[8]报道了15例CD34^+^细胞纯化输注治疗无效的患者，3例存活，12例死亡，主要的死亡原因是感染并发症。本文例3患者CD34^+^细胞纯化输注治疗后2个月评估为NR，输血依赖，病程后期因重症感染死亡。

Cuadrado等[8]的研究报道，CD34^+^细胞纯化输注后的aGVHD发生率为11％（7例），其中1～2度3例，3～4度4例；cGVHD发生率为8％（5例），轻度1例，中度1例，重度3例。尚无MF患者CD34^+^细胞纯化输注治疗后GVHD情况的大样本研究。本文3例患者在接受CD34^+^细胞纯化输注前均已停用钙调磷酸酶抑制剂，考虑到采集的供者干细胞经Miltenyi磁珠CD34^+^细胞分选技术处理后，已经实现了T细胞去除，故3例患者在CD34^+^细胞纯化输注前未行预处理，随访期间均无aGVHD表现。其中1例患者CD34^+^细胞纯化输注后195 d出现口腔cGVHD，经西罗莫司治疗后缓解。还需积累更多病例进一步观察CD34^+^细胞纯化输注后MF患者GVHD的发病情况及其对患者生存的影响。

研究显示，大多数患者血细胞计数恢复的中位时间为230（23～1 146）d，对于移植后未进行治疗的PGF患者，24个月内也有近40％的患者血象改善[9]。Oyekunle等[10]报道的3例MF患者CD34^+^细胞纯化输注时间分别为造血干细胞移植后118 d、123 d和132 d。法国一项多中心研究纳入55例接受CD34^+^细胞纯化输注治疗者，启动治疗的中位时间为造血干细胞移植后119（78～222）d[11]。Fraint等[12]报道的14例儿童启动CD34^+^细胞纯化输注治疗的中位时间为造血干细胞移植后148（50～431）d。本文3例PMF患者分别于移植后56 d、154 d和83 d接受了CD34^+^细胞纯化输注治疗。本研究组认为血细胞减少的程度和持续时间是启动CD34^+^细胞纯化输注治疗的决定因素，对于PMF患者，allo-HSCT后2～3个月若造血功能未恢复，即筹划CD34^+^细胞纯化输注治疗。例2因治疗HHV-6感染和等待骨髓库无关供者二次捐献造血干细胞准备期长，故CD34^+^细胞纯化输注启动时间较晚。

CD34^+^细胞纯化输注能有效治疗PGF，且未观察到严重的GVHD。未来仍有待探索更优化的预后模型、通过临床指标或生物学标志物识别出预后不良的PGF患者，以确定启动CD34^+^细胞纯化输注等干预措施的理想时机，或将CD34^+^细胞纯化输注提前至造血干细胞移植后5 d进行PGF预防[13]。
